# Biodiversity of macrophyte communities and associated aquatic organisms in lakes of the Vologda Region (north-western Russia)

**DOI:** 10.3897/BDJ.10.e77626

**Published:** 2022-01-20

**Authors:** Dmitriy A. Philippov, Ksenya N. Ivicheva, Nadezhda N. Makarenkova, Igor V. Filonenko, Aleksandra S. Komarova

**Affiliations:** 1 Papanin Institute for Biology of Inland Waters Russian Academy of Sciences, Borok, Russia Papanin Institute for Biology of Inland Waters Russian Academy of Sciences Borok Russia; 2 Vologda branch of the Russian Federal Research Institute of Fisheries and Oceanography, Vologda, Russia Vologda branch of the Russian Federal Research Institute of Fisheries and Oceanography Vologda Russia

**Keywords:** Russia, Eastern Europe, Vologda Region, Vozhe Lake, Kubenskoe Lake, small lakes, macrophytes, microalgae, aquatic invertebrates, occurrences, sampling event, dataset, data paper, rare species

## Abstract

**Background:**

This paper provides current data on the biodiversity of boreal lakes of the Vologda Region (north-western Russia), including macrophytes (vascular plants and macroscopic algae) and macrophyte inhabitants (invertebrates and microalgae). The raw data, given in two datasets (Sampling event dataset and an Occurrence dataset) and presented in the form of GBIF-mediated data, were collected from 139 lakes (macrophytes between 2005 and 2021, macrophyte inhabitants between 2014 and 2020). The dataset contains materials on the diversity of vascular plants (Tracheophyta, 3225 occurrences; Bryophyta, 155; Marchantiophyta, 16), macro- and microalgae (Ochrophyta, 546 occurrences; Chlorophyta, 193; Charophyta, 153; Cyanobacteria, 139; Cryptophyta, 86; Myzozoa, 33; Euglenozoa, 27; Rhodophyta, 8; Bigyra, 1) and aquatic invertebrates (Arthropoda, 1408 occurrences; Annelida, 487; Mollusca, 263; Platyhelminthes, 36; Cnidaria, 11). This paper summarises previously unpublished materials in a standardised form.

**New information:**

The paper summarises the data collected during the long-term phytodiversity studies in a series of lakes of different types (Vologda Region, north-western Russia). Data on algae and invertebrates diversity were obtained in 60 different plant communities of aquatic, semi-aquatic and coastal plants or their combinations. A total of 6787 occurrences were included in the dataset, published in the global biodiversity database (GBIF) for the first time. According to the GBIF taxonomic backbone, the dataset comprised 837 taxa, including 711 lower-rank taxa (species, subspecies, varieties, forms). New records of 47 species rare and protected in the Vologda Region are given: 43 species of plants, three species of animals and one species of Cyanobacteria.

## Introduction

The studies of macrophytes in lakes of the Vologda Region have a relatively short history (Philippov 2010). In the 1960s and 70s, beyond the local studies of individual lakes, two large complex limnological expeditions occurred. The first expedition ("ozernaya ekspeditsiya") operated from 1969 to 1975 and managed to study 275 small lakes located mainly in the western part of the Vologda Region. It was organised by the Vologda State Pedagogical Institute and led by ichthyologist Lev A. Zhakov (later, continued by German A. Vorobyev). The main results of this expedition were presented in reports and a few publications (Vorobyev 1973, Vorobyev 1977, Lyapkina and Shevelev 1981). Studies of macrophytes and their communities were carried out by Roman V. Bobrovskiy or under his supervision; however, the main results remained unpublished or were presented in a very brief form (Philippov et al. 2019). The second major expedition ("Vologodsko-Arkhangelskaya ekspeditsiya") was organised by the Institute of Limnology of the Academy of Sciences USSR. Its leader was Igor M. Raspopov, who was also responsible for macrophyte research. In 1972-1974, Kubenskoe Lake and Vozhe Lake were studied and, in 1974-1977, Beloe Lake. Later, the results of botanical research were summarised (Raspopov 1985).

Our studies of macrophytes were carried out between 2005 and 2021. Both large, Kubenskoe Lake and Vozhe Lake and a long series of small lakes of various sizes and genesis were studied. Some data on the macrophytes in the lakes of the Vologda Region were published previously in the research articles (Philippov and Czhobadze 2015, Sadokov and Philippov 2017, Chernova et al. 2019) and short notes on the findings of macrophyte species, rare or new to the area (Afonina et al. 2010, Abolin et al. 2011, Chemeris et al. 2011, Bobrov and Philippov 2012, Sofronova et al. 2012, Sofronova et al. 2017, Sofronova et al. 2018, Philippov et al. 2016, Leostrin et al. 2018, Vishnyakov and Philippov 2018, Levashov et al. 2019, Vishnyakov et al. 2021). Finally, macrophyte data were summarised in the GBIF occurrence dataset (Philippov and Komarova 2021).

Macrophytes are an essential habitat for invertebrates and algae. Invertebrates utilise aquatic plants as a direct food source (Gregg and Rose 1982, Gregg and Rose 1985), shelter from predators (Harrod 1964), spawning grounds (Keast 1984), attachment sites or feeding grounds in the case of periphyton-consuming animals (Cattaneo et al. 2008). Therefore, the composition and development of macrophyte communities, as well as architecture of their leaves, the growth habit of the plant and the presence of chemical inhibitors in the plant tissue may affect the invertebrate abundance, diversity and community composition (Talbot and Ward 1987, Balci and Kennedy 2003). The studies of macrophyte communities' inhabitants in the Vologda Region are scarce (Philippov 2010); therefore, the data we collected and summarised in the sampling event dataset (Philippov et al. 2021) have scientific novelty and are of considerable interest to hydrobiologists.

## Project description

### Title

Diversity, distribution, ecology, biology of aquatic and semi-aquatic plants in the European North

### Personnel

Dmitriy A. Philippov

## Sampling methods

### Study extent

A list of records of macrophytes and macrophyte inhabitants (invertebrates and microalgae) in lakes of the Vologda Region is presented. By macrophytes, we understood macroscopic plants, regardless of their taxonomic position and ecological characteristics. Macrophytes include vascular plants, mosses, liverworts and large multicellular algae (Papchenkov et al. 2003). We determined the flora of lakes as aquatic species and species directly related to the aquatic environment (helophytes, plants of the water’s edge, amphibious plants, hygrophytes, plants of drying sandbanks).

### Sampling description

Field studies were carried out from June to October, mainly during the greatest development of macrophytes (July and August). The composition of the flora of lakes was established during route field studies. We studied all accessible microhabitats in the lakes and their coastal parts, including those differing in current velocity, sediments, depths and macrophyte canopy development. When investigating small lakes, from 0.05 to 0.1 km^2^, a route was made by walking around a lake or going around by boat along the entire coastline. In larger lakes, floristic studies were conducted at several reference sites, located mainly in highly-developed macrophyte communities. For hydrobiological studies, sampling was performed at model sites only.

### Quality control

The data were collected and identified by scientists from the Papanin Institute for Biology of Inland Waters Russian Academy of Sciences and the Vologda Branch of the Russian Federal Research Institute of Fisheries and Oceanography. The accuracy of the determination of some samples was confirmed by systematics from the Institute of Biology of Komi Scientific Centre of the Ural Branch of the Russian Academy of Sciences, Institute of Biology of Karelian Research Centre of the Russian Academy of Sciences (Russian Federation) and the University of Warmia and Mazury in Olsztyn (Poland).

### Step description

Research problem formulation.

Logistic issues resolution, including the choice of routes, water objects, time and duration of work.

Field stage: obtaining samples and other original materials on the diversity of macrophytes, aquatic algae and invertebrates.

(a) Macrophytes. In the field, pictures of plants and floristic lists were made, some species were collected in a herbarium; several hydrochemical parameters (water temperature, total dissolved solids, pH and electrical conductivity) were measured using portable devices (Philippov et al. 2017).

(b) Algae. Samples were taken with a 1-litre Patalas bathometer from three layers of the water column in macrophyte communities. For microalgae sedimentation, water samples were treated with Lugol's iodine solution for 10–14 days to obtain the final volume of 25 ml (Kuzmin 1975).

(c) Aquatic invertebrates. The study of invertebrates in macrophyte communities was conducted by preparing washed-off samples from plants (Mitropolskiy and Mordukhai-Boltovskoi 1975b) and by sampling sediments in the same communities (Mitropolskiy and Mordukhai-Boltovskoi 1975a). Sediment sampling was carried out from a boat by a three-times lifting of a GR-91 rod bottom-grab (sampling area 0.007 m^2^) or a one-time lifting of the Petersen dredge (sampling area 0.025 m^2^). At each sampling site, sediment samples were washed straight away through a sieve with a 250 μm mesh. After that, sediment samples were placed in plastic containers and preserved in 40% formaldehyde solution. Epiphyton samples (zoophytos) collection was slightly different from one macrophyte communities to the others. Submerged aquatic plants and aquatic plants with floating leaves were removed from the water, placed in a nylon sieve and washed out of all macroinvertebrates. In a sieve (250 µm mesh), all macroinvertebrates were separated from the plant substrate by rinsing and mechanical separation; then plants were dried from moisture and weighed. In helophytes and hygrohelophytes, a part of plants submerged in water was used for analysis. The underwater part was first placed in a nylon sieve and washed, then weighed. Semi-aquatic plants (including those from floating mats) were taken from plots of 25 × 25 cm^2^; when sampling vascular plants, the entire overground part of a plant was cut off; when sampling mosses, the whole moss clumps were taken and placed in a sieve. After washing off, samples of invertebrates (sometimes with fragments of macrophytes) were placed in plastic containers and fixed with 40% formaldehyde solution. Aquatic mosses were placed in plastic containers without rinsing with water and fixed with 40% formaldehyde solution.

Data collection: analysis of samples not identified in the field or verification of the identification data by the experts.

(a) Macrophytes. The keys by Tzvelev (2000), Ignatov and Ignatova (2003), Ignatov and Ignatova (2004) and Lisitsyna et al. (2009) were used in the study. Herbarium materials were transferred for processing to the Herbarium of the Mire Research Group of Papanin Institute for Biology of Inland Waters Russian Academy of Sciences (MIRE).

(b) Algae. Sedimented phytoplankton for qualitative and quantitative analysis was examined in a Nageotte counting chamber (0.01 cm^3^) using a Mikmed-6 microscope (LOMO, Russia) at 640× magnification. The biomass of microalgae was calculated using direct counts of the volumes equated to geometric figures of cells. The specific weight of algae was conditionally taken equal to one (Kuzmin 1975). For damaged cells which were not used for the biomass count, a value of 1 was assigned in the column Organism quantity. Taxonomic identification was made to the closest possible low-rank taxon using all keys and summaries available: Kiselev (1954), Ettl (1978), Komárek and Fott (1983), Starmach (1985), Krammer and Lange-Bertalot (1986), Krammer and Lange-Bertalot (1988), Krammer and Lange-Bertalot (1991a), Krammer and Lange-Bertalot (1991b), Komárek and Anagnostidis (1998), Komárek and Anagnostidis (2005), Palamar-Mordvintseva (2003), Vetrova (2004), Coesel and Meesters (2007), Komárek (2013) etc.

(c) Aquatic invertebrates. All specimens were identified with an MBS-10 stereoscopic microscope and a Mikmed-6 microscope (LOMO, Russia) using all keys and summaries available: Kutikova and Starobogatov (1977), Tsalolikhin (1994), Tsalolikhin (2001), Tsalolikhin (2016), Narchuk et al. (1997), Narchuk and Tumanov (2000)etc. Specimens of each species were dried with filter paper and weighed using Shimadzu AUX-120 scales (Japan) with 0.0001 g accuracy. Moss mats were cleared of all invertebrates, dried on filter paper and weighed. Quantity and biomass counts of sediment-associated invertebrates were made by 1 m^2^ (g/m^2^). In washed-off samples, quantity and biomass counts were made by 1 kg of macrophyte wet weight (g/kg).

Records list compilation. The dataset fields’ names were chosen according to Darwin Core (Wieczorek et al. 2012) and include the following: «occurrenceID», «basisOfRecord», «scientificName», «eventID», «eventDate», «taxonRank», «kingdom», «phylum», «class», «order», «family», «genus», «habitat», «samplingProtocol», «sampleSizeValue», «sampleSizeUnit», «individualCount», «organismQuantity», «organismQuantityType», «decimalLatitude», «decimalLongitude», «geodeticDatum», «coordinateUncertaintyInMetres», «coordinatePrecision», «countryCode», «country», «stateProvince», «county», «locality», «year», «month», «day», «recordedBy», «identifiedBy», «dateIdentified», «associatedReferences». Georeferencing was made using a GPS navigator or Google maps. For macrophytes, coordinates accuracy was maintained in a 30–250 m range, rarely greater; for other groups of aquatic organisms, 50 m. Coordinates were determined to the fourth digit. In all cases, the WGS-84 coordinate system was used.

## Geographic coverage

### Description

**Figure 1.**
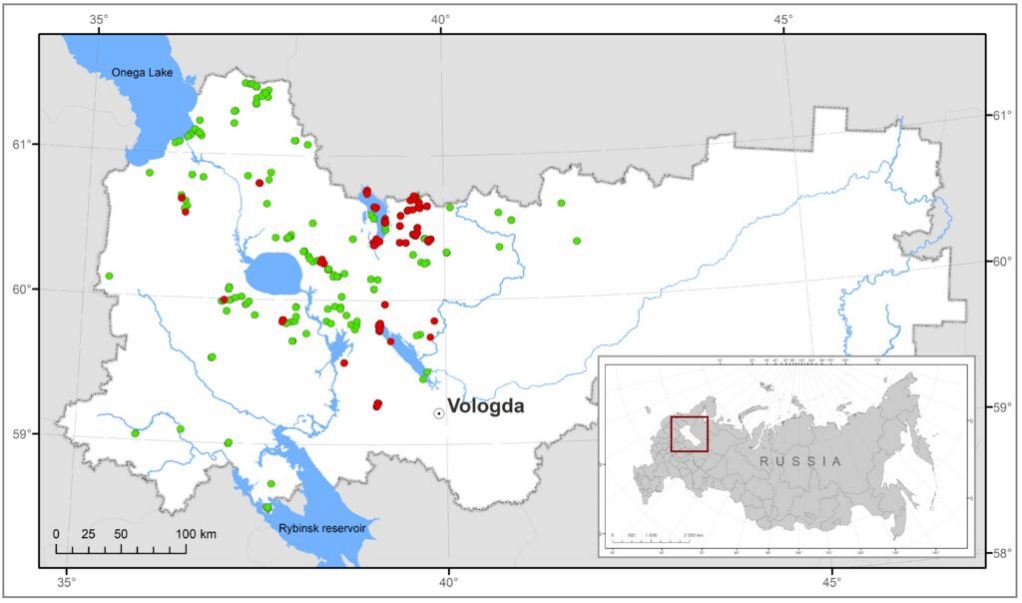
Study area and sampling localities. Occurrences of macrophytes in lakes are shown as green circles, other aquatic organisms – red circles.

Vologda Region is situated in the north-western part of Russia within the northern part of the East European Plain (Fig. 1). The length of the region from the north to the south is 350 km (N 58°29', N 61°35'), from west to east – 700 km (E 34°43', E 47°09'). The area of the Vologda Region is 145.7 km^2^. The Region is located on the border of the southern and middle taiga subzones. The ground surface heights vary from 33 to 304 m above sea level; therefore, the morphological complexes of lowlands, medium-altitude plains and low elevations can be found in the Region (Vorobyev 2007).

The hydrographic network of the Region is very diverse. About 20 thousand watercourses flow in the Region, belonging to three basins of global flow: the White Sea (70% of basin area), the Baltic Sea (8%) and the Caspian Sea (22%) (Filenko 1966). Several water reservoirs were built in the Vologda Region; Rybinsk Reservoir and Sheksna Reservoir are the largest and well-known (Vorobyev 2007). The Region is significantly paludified; more than 17% of the area is covered with mires of various types (Filonenko and Philippov 2013).

There are over five thousand lakes in the Vologda Region, most located in its western part. In the north-western districts of the Region, the total area of lakes in a district ranges from 3% to 10% of the district’s area; to the east and southeast of the border of the last glaciation, the indices do not exceed 2% and, in some eastern districts of the region, it is only a fraction of a percent. The total area of lakes in the region is 4.3 thousand km^2^ or about 3% of the Region’s territory. A relatively small number of lakes (only 25) with a water surface of more than 10 km^2^ comprise 84% of the total area of lakes. Lakes of glacial-tectonic origin (Lakes Onega, Beloe, Vozhe and Kubenskoe) make up this group of lakes. The absolute majority of lakes are small (water surface area less than 0.1 km^2^). Lakes with a water surface area of 0.01 to 0.1 km^2^ account for 5.5% of the total area of lakes in the Region. The group of small lakes includes forest drainless lakes, floodplain oxbow lakes, intra-mire lakes and karst lakes (Antipov 1981).

The main reason for such a distribution of lakes across the Region is the time since the glaciation. The north-western areas of the Region, later freed from the glacier, retained the features of young relief with numerous inter-hill and inter-ridge depressions, which were filled with glacial waters. As the glacier retreated, thaw waters formed periglacial and postglacial reservoirs in the depressions. Following a decrease in the water level and vegetation development in water bodies, some of them turned into vast paludified lowlands (for example, the Mologo-Sheksninskaya lowland). Other water bodies have significantly decreased in size, but remained in the lowlands in the form of vestigial shallow lakes (Vorobyev 1973).

Most of the lakes in the Region are shallow. Relict water bodies of glacial-lake plains have shallow depths (for example, the average depth of Lake Vozhe is 1.8 m, Lake Kubenskoe 2.5 m). The deepest lakes are located in moraine-hilly landscapes: Lake Sodoshnoe (40 m), Lake Ferapontovskoe (27 m), Lake Siverskoe (26 m) and Lake Svyatoe (25 m). A thermal regime with distinct direct temperature stratification in summer and reverse stratification in winter is observed only in the deepest lakes. These lakes are characterised by the highest values of the heat budget (5–7 kcal/cm^2^) and the temperature of the bottom water layer is below 10°С in summer. Lakes with unclear and unstable stratification, a bottom temperature above 10–15°C and a lower heat budget are much more common. The beginning of lake ice-covering usually falls in the first third of November. As a rule, the opening occurs in the first third of May. The lakes are covered in ice for 160–175 days on average, usually longer than rivers (Filenko 1966, Antipov 1981, Vorobyev 2007).

All the lakes in the Vologda Region are freshwater lakes with TDS values within the zonal norm, of bicarbonate-calcium composition as a rule. Mostly, lake waters are neutral or slightly alkaline (pH 6.9–7.5), favourable for aquatic organisms (Vorobyev and Korobeynikova 1981). On the other hand, intra-mire lakes have a wide pH range, more often slightly acidic or acidic (pH 4.2–6.5) (Komov and Stepanova 1994, Philippov and Yurchenko 2020).

Lakes in the Vologda Region have a different degree, character and intensity of macrophyte covering, closely related to landscape and limnological conditions (Vorobyev 1977, Sadokov and Philippov 2017).

Photographs of some studied lakes and macrophyte communities are given below (Figs 2, 3, 4, 5, 6, 7, 8, 9, 10, 11, 12, 13, 14).

### Coordinates

59.264 and 60.804 Latitude; 36.305 and 39.849 Longitude.

## Taxonomic coverage

### Description

This dataset provides current data on vascular plants, cryptogams, microalgae and aquatic invertebrates in lakes of the Vologda Region. The list contains records on Animalia (5 phyla, 7 classes, 22 orders, 64 families), Bacteria (1 phylum, 1 class, 4 orders, 11 families), Chromista (4 phyla, 7 classes, 28 orders, 40 families), Plantae (6 phyla, 15 classes, 48 orders, 81 families) and Protozoa (1 phylum, 1 class, 1 order, 2 families) species. Overall, the dataset comprises 837 taxa, including 711 lower-rank taxa (species, subspecies, varieties, forms).

### Taxa included

**Table:** 

Rank	Scientific Name	
kingdom	Animalia	
kingdom	Bacteria	
kingdom	Chromista	
kingdom	Plantae	
kingdom	Protozoa	

## Traits coverage

### Data coverage of traits

PLEASE FILL IN TRAIT INFORMATION HERE

## Temporal coverage

### Notes

2005 to 2021

## Usage licence

### Usage licence

Other

### IP rights notes

This work is licensed under a Creative Commons Attribution (CC-BY) 4.0 License.

## Data resources

### Data package title

Data on the biodiversity of macrophyte communities and associated aquatic organisms in lakes of the Vologda Region (north-western Russia)

### Resource link

https://www.gbif.org/dataset/6f0d0430-b719-4a2e-9bb0-20b35ba4bc6c;https://www.gbif.org/dataset/a78dcaca-c58f-4525-a0de-76b7763f7a9f

### Alternative identifiers

http://gbif.ru:8080/ipt/resource?r=macrophyte-vologda;http://gbif.ru:8080/ipt/resource?r=macrophytes-vologda-occurrences

### Number of data sets

2

### Data set 1.

#### Data set name

Data on the biodiversity of macrophyte communities and associated aquatic organisms in lakes of the Vologda Region (North-Western Russia): algae and invertebrates

#### Data format

Darwin Core

#### Number of columns

38

#### Character set

Sampling event dataset

#### Download URL

https://www.gbif.org/dataset/6f0d0430-b719-4a2e-9bb0-20b35ba4bc6c

#### Data format version

1.4

#### Description

This dataset provides current data on the biodiversity of boreal lakes of the Vologda Region (orth-western Russia), including macrophytes (vascular plants and macroscopic algae) and macrophyte inhabitants (invertebrates and microalgae). The data were collected from 139 lakes. The dataset contains materials on the diversity of vascular plants (Tracheophyta, 3225 occurrences; Bryophyta, 155; Marchantiophyta, 16), macro- and microalgae (Ochrophyta, 546 occurrences; Chlorophyta, 193; Charophyta, 153; Cyanobacteria, 139; Cryptophyta, 86; Myzozoa, 33; Euglenozoa, 27; Rhodophyta, 8; Bigyra, 1) and aquatic invertebrates (Arthropoda, 1408 occurrences; Annelida, 487; Mollusca, 263; Platyhelminthes, 36; Cnidaria, 11). A total of 6787 occurrences are included in the list.

**Data set 1.** 

Column label	Column description
eventID	Identifier of the event, unique for the dataset (MiReGr_Alg_xxx_SmLake_sxxxx; MiReGr_Alg_xxx_BigLake_sxxxx; MiReGr_Zoo_xxx_SmLake_xxxx; MiReGr_Zoo_xxx_BigLake_xxxx).
occurrenceID	Identifier of the record, coded as a global unique identifier.
eventDate	The date or interval during which an event occurred. For occurrences, this is the date when the event was recorded. A variable.
samplingProtocol	Reference with description of the method or protocol used during a sampling event. A variable (three options: “Kuzmin GV (1975) Phytoplankton. Species composition and abundance. In: Mordukhai-Boltovskoi PhD (Ed.) Methodology for the study of biogeocenoses of inland waters. Nauka, Moscow, 73-87 pp.”; “Mitropolskiy VI, Mordukhai-Boltovskoi PhD (1975) Makrozoobenthos. In: Mordukhai-Boltovskoi PhD (Ed.) Methodology for the study of biogeocenoses of inland waters. Nauka, Moscow, 158-170 pp.”; “Mitropolskiy VI, Mordukhai-Boltovskoi PhD (1975b) Biofouling, phytophilic biocenoses and planktobenthos. In: Mordukhai-Boltovskoi PhD (Ed.) Methodology for the study of biogeocenoses of inland waters. Nauka, Moscow, 171-178 pp.”).
sampleSizeValue	A numeric value for a measurement of the area, weight or volume of a sample in a sampling event. A variable.
sampleSizeUnit	The unit of measurement of the size of a sample in a sampling event. A variable (three options: “kilogram”; “litre”; “m^2^”).
decimalLatitude	The geographic latitude in decimal degrees of the geographic centre of the data sampling place.
decimalLongitude	The geographic longitude in decimal degrees of the geographic centre of the data sampling place.
geodeticDatum	The ellipsoid, geodetic datum or spatial reference system (SRS) upon which the geographic coordinates given in decimalLatitude and decimalLongitude are based. A constant ("WGS84").
coordinateUncertaintyInMetres	The maximum uncertainty distance in metres.
countryCode	The standard code for the Russian Federation according to ISO 3166-1-alpha-2 (RU).
country	Country name (Russian Federation).
stateProvince	Region (‘oblast’) name. The first-level administrative division. A constant ("Vologda Region").
county	District (‘rayon’) name. The second-level administrative division.
locality	The specific description of the place. This term may contain information modified from the original to correct perceived errors or to standardise the description. A variable (names of lakes).
habitat	A category or description of the habitat in which the Event occurred, in Russian.
year	The four-digit year in which the Event occurred, according to the Common Era Calendar.
month	The integer month in which the Event occurred.
day	The integer day of the month on which the Event occurred.
basisOfRecord	The specific nature of the data record in standard label of one of the Darwin Core. A constant ("PreservedSpecimen").
scientificName	The full scientific name, with authorship and date information, if known.
taxonRank	The taxonomic rank.
kingdom	The full scientific name of the kingdom in which the taxon is classified.
phylum	The full scientific name of the phylum or division in which the taxon is classified.
class	The full scientific name of the class in which the taxon is classified.
order	The full scientific name of the order in which the taxon is classified.
family	The full scientific name of the family in which the taxon is classified.
genus	The full scientific name of the genus in which the taxon is classified.
individualCount	The number of individuals represented present at the time of the Occurrence.
organismQuantity	Number or enumeration value for the quantity of organisms.
organismQuantityType	The type of quantification system used for the quantity of organisms. A variable (three options: “mg/l”; “g/kg”; “g/m^2^”).
recordedBy	List of persons who collected field data.
identifiedBy	A person who assigned the Taxon to the subject.
dateIdentified	The date when the taxonomic identification happened.
language	A language of the resource (en | ru).
acceptedNameUsage	The full name, with authorship and date information, if known, of accepted taxon.
taxonomicStatus	The taxonomic status of a taxon. A variable (accepted or synonym).
taxonRemarks	Remarks regarding taxa.

### Data set 2.

#### Data set name

Data on the biodiversity of macrophyte communities and associated aquatic organisms in lakes of the Vologda Region (North-Western Russia): macrophytes

#### Data format

Darwin Core

#### Number of columns

31

#### Character set

Occurrence dataset

#### Download URL

https://www.gbif.org/dataset/a78dcaca-c58f-4525-a0de-76b7763f7a9f

#### Data format version

1.2

**Data set 2.** 

Column label	Column description
occurrenceID	An identifier for the record, unique within this dataset. An abbreviation in the identifier' number (MiReGr_LakeBioDiv_xxxxx).
basisOfRecord	The specific nature of the data record in standard label of one of the Darwin Core. A constant ("HumanObservation").
scientificName	The full scientific name, with authorship and date information, if known.
eventDate	The date or interval during which an event occurred. For occurrences, this is the date when the event was recorded. A variable.
taxonRank	The taxonomic rank.
kingdom	The full scientific name of the kingdom in which the taxon is classified.
phylum	The full scientific name of the phylum or division in which the taxon is classified.
class	The full scientific name of the class in which the taxon is classified.
order	The full scientific name of the order in which the taxon is classified.
family	The full scientific name of the family in which the taxon is classified.
genus	The full scientific name of the genus in which the taxon is classified.
habitat	A category or description of the habitat in which the Event occurred.
decimalLatitude	The geographic latitude in decimal degrees of the geographic centre of the data sampling place.
decimalLongitude	The geographic longitude in decimal degrees of the geographic centre of the data sampling place.
geodeticDatum	The ellipsoid, geodetic datum or spatial reference system (SRS) upon which the geographic coordinates given in decimalLatitude and decimalLongitude are based. A constant ("WGS84").
coordinateUncertaintyInMetres	The maximum uncertainty distance in metres.
coordinatePrecision	A decimal representation of the precision of the coordinates given in the decimalLatitude and decimalLongitude. A constant ("0.0001").
countryCode	The standard code for the Russian Federation according to ISO 3166-1-alpha-2 (RU).
country	Country name (Russian Federation).
stateProvince	Region (‘oblast’) name. The first-level administrative division. A constant ("Vologda Region").
county	District (‘rayon’) name. The second-level administrative division.
locality	The specific description of the place. This term may contain information modified from the original to correct perceived errors or to standardise the description. A variable (names of lakes).
year	The four-digit year in which the Event occurred, according to the Common Era Calendar.
month	The integer month in which the Event occurred.
day	The integer day of the month on which the Event occurred.
recordedBy	List of persons who collected field data.
identifiedBy	A person who assigned the Taxon to the subject.
dateIdentified	The date when the taxonomic identification happened.
associatedReferences	List of literature references associated with the occurrences.
acceptedNameUsage	The full name, with authorship and date information, if known, of accepted taxon.
taxonomicStatus	The taxonomic status of a taxon. A variable (accepted or synonym).

## Additional information

The paper provides materials on the diversity of vascular plants, macro- and microalgae and aquatic invertebrates. A total of 6787 occurrences are included in the list: Tracheophyta, 3225 occurrences; Bryophyta, 155; Marchantiophyta, 16; Ochrophyta, 546; Chlorophyta, 193; Charophyta, 153; Cyanobacteria, 139; Cryptophyta, 86; Myzozoa, 33; Euglenozoa, 27; Rhodophyta, 8; Bigyra, 1; Arthropoda, 1408; Annelida, 487; Mollusca, 263; Platyhelminthes, 36; Cnidaria, 11.

Based on 3464 occurrences (taxon per sampling site), the flora of lakes in the Vologda Region is represented by 243 low-rank taxa of macrophytes from 129 genera, 67 families, 40 orders, 11 classes and six phyla.

Macrophytes belong to different ecological groups, which can be combined into three ecotype groups: aquatic plants, semi-aquatic plants and coastal plants. Samples were collected both in macrophyte communities belonging to one ecotype group and in complex communities comprised of macrophytes from different ecotype groups. For the latter, a "macrophyte combinations" category was assigned.

**Figure 15.**
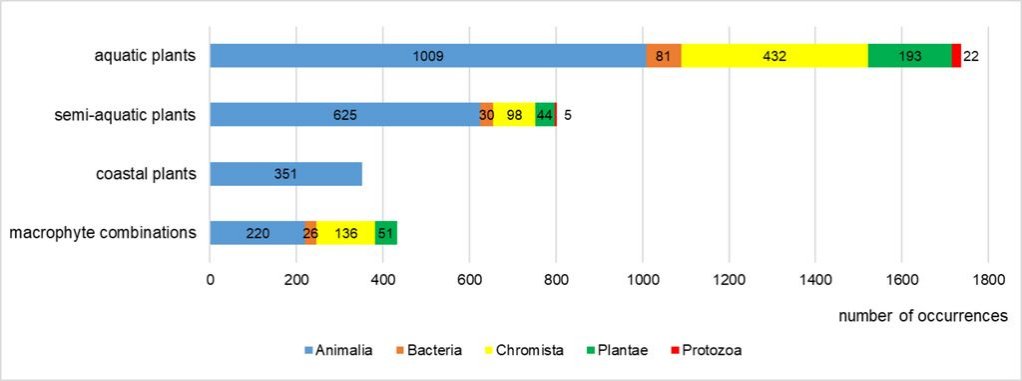
Number of occurrences of aquatic organisms inhabiting the macrophyte communities of different ecotype groups.

The largest number of occurrences came from aquatic plants (1737 occurrences), with the highest values of occurrences of representatives of each kingdom (Fig. 15). Slightly fewer occurrences were in the communities of semi-aquatic plants (802) and macrophyte combinations (433). Finally, the smallest number of occurrences came from the communities of coastal plants (351).

**Figure 16.**
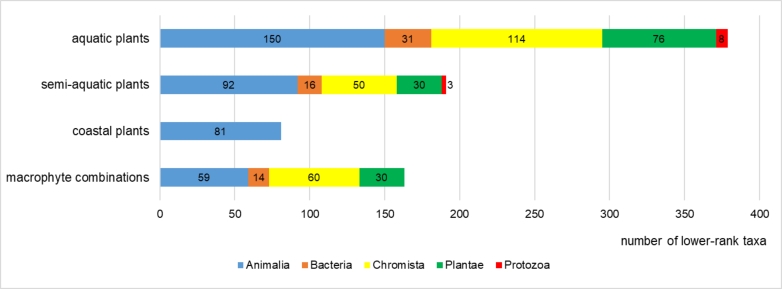
Number of lower-rank taxa of aquatic organisms inhabiting the macrophyte communities of different ecotype groups.

The greatest number of lower-rank taxa (species, subspecies, variety, form) belonged to aquatic plants (379; Fig. 16). Similar values of lower-rank taxa came from the communities of semi-aquatic plants (191) and macrophyte combinations (163). The smallest amount of lower-rank taxa were found in coastal plants communities (81). The highest values of lower-rank taxa of each kingdom were registered in the communities of aquatic plants.

Within each ecotype group, we investigated several ecological groups of macrophytes, based on the classification proposed by V.G. Papchenkov (2001). In our studies, samples were taken both in monodominant and complex communities. Occurrences distribution through different macrophyte communities, ecological groups and ecotype groups is given below (Table 1). The greatest number of occurrences came from communities of aquatic plants: *Nupharlutea* (297), *Fontinalisantipyretica* (243), *Persicariaamphibia* (197), *Potamogetonnatans* (174), *Scorpidiumscorpioides* (126), *Potamogetonlucens* (104), semi-aquatic plants: *Phragmitesaustralis* (269), *Schoenoplectuslacustris* (123) and coastal plants: *Sphagnum* sp. (102).

In the studied lakes, 47 rare and protected species of plants, animals and Cyanobacteria were found. Amongst them, a stonewort (*Charastrigosa*), two species of quillworts (*Isoetesechinospora*, *Isoeteslacustris*) and a dragonfly (*Anaximperator*) are listed in the Red Data Book of the Russian Federation (Danilov-Danilyan 2001, Bardunov and Novikov 2008); 23 species are listed in the Red Data Book of the Vologda Region (Bolotova et al. 2010, Suslova et al. 2013, Anonymous 2015): Critically Endangered (CR) - *Cornussuecica*; Endangered (EN) - *Carexbuxbaumii*, *Lobeliadortmanna*, *Potamogetoncrispus*; Vulnerable (VU) - *Batrachiumcircinatum*, *Myriophyllumalterniflorum*, *Nostocpruniforme*, *Nupharpumila*, *Nymphaeatetragona*, *Ricciacanaliculata*; Near Threatened (NT) - *Droseraanglica*, *Moliniacaerulea*, *Potamogetonfriesii*, *Rhynchosporaalba*, *Trichophorumalpinum* [as *Baeothryonalpinum* (L.) Egor.], *Utriculariaminor*; Least Concern (LC) - *Carexpseudocyperus*, *Charavirgata*, *Ligulariasibirica*, *Moerckiaflotoviana* [as *M.hibernica* (Hook.) Gottsche], Seneciopaludosussubsp.lanatus Holub [as *S.tataricus* Less.]; Data Deficient (DD) - *Alismagramineum*, *Dytiscuslatissimus*; another 20 species are rare in the Region and assigned with the status “biological control required”: *Batrachiumtrichophyllum*, *Betulahumilis*, *Charatomentosa*, *Gammaruslacustris*, *Harpanthusflotovianus*, *Hydrocharismorsus-ranae*, *Irispseudacorus*, *Nitellaflexilis*, *Nymphaeacandida*, *Potamogetonberchtoldii*, *Potamogetonfiliformis*, *Potamogetonpraelongus*, *Rumexhydrolapathum*, *Salixlapponum*, *Scolochloafestucacea*, *Sparganiumangustifolium*, *Sparganiumnatans*, *Stratiotesaloides*, *Typhaangustifolia* and *Utriculariaintermedia*.

## Figures and Tables

**Figure 2.**
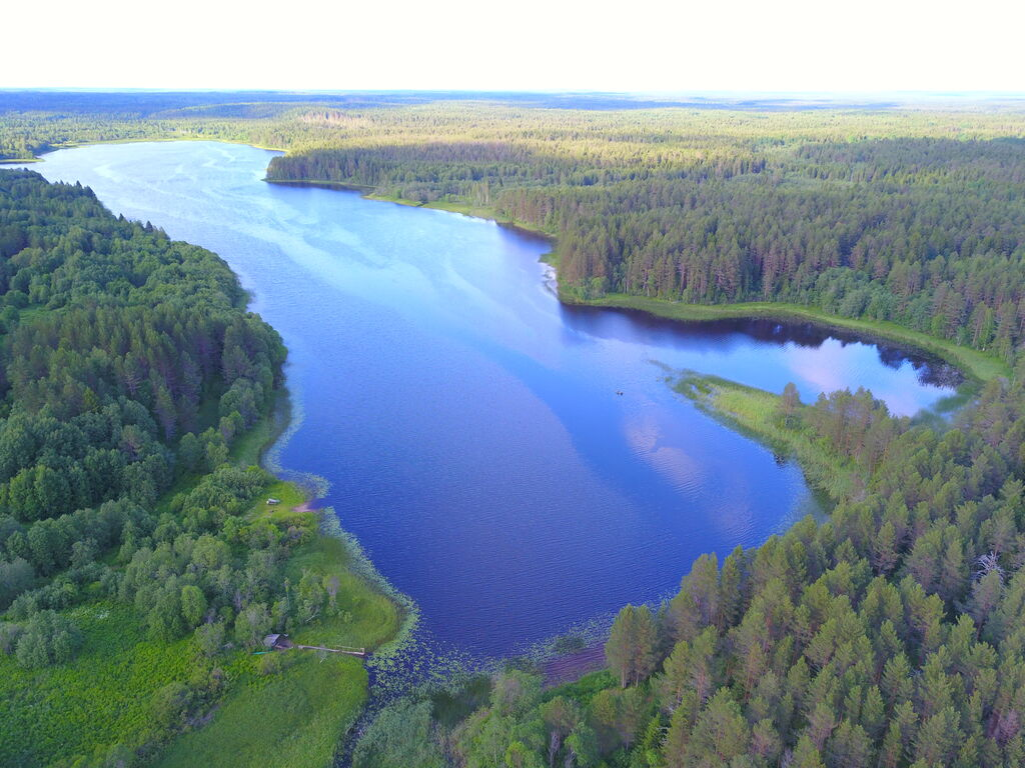
An example of a lake with a forested drainage basin, Svyatoe lake (Vologda Region, Russia). Photo by Dmitriy A. Philippov (2019).

**Figure 3.**
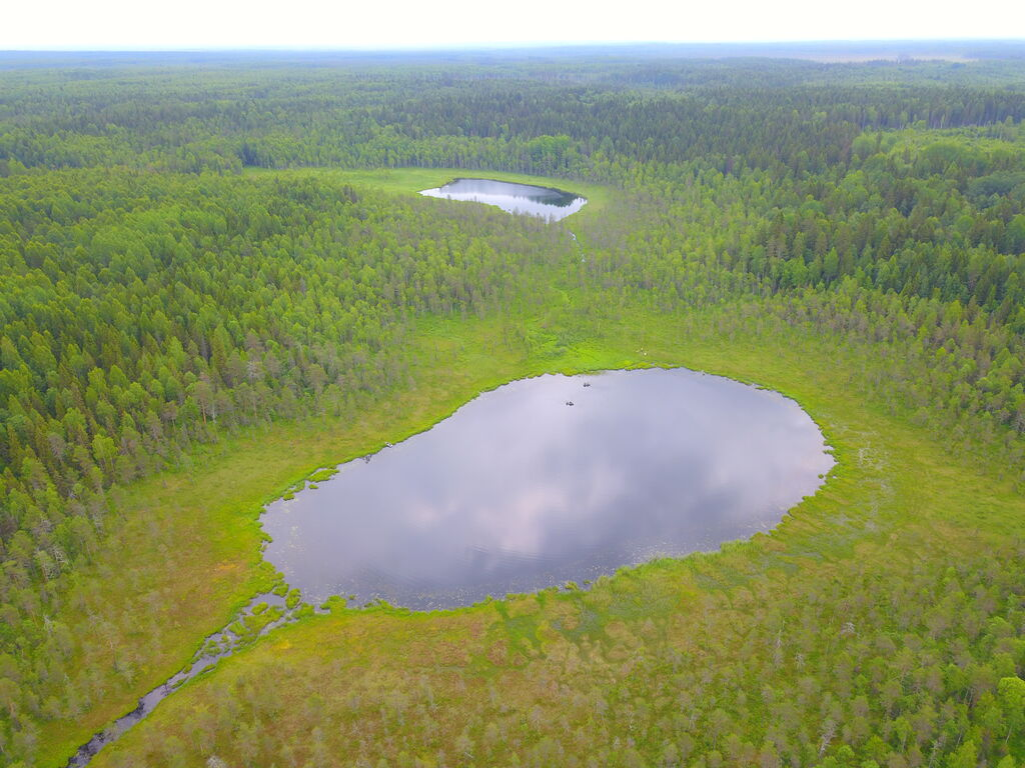
An example of small intra-mire lakes connected by a river, Lapovskoe-2 Lake (background), Lapovskoe-1 Lake (foreground) and Lapovka River (Vologda Region, Russia). Photo by Dmitriy A. Philippov (2020).

**Figure 4.**
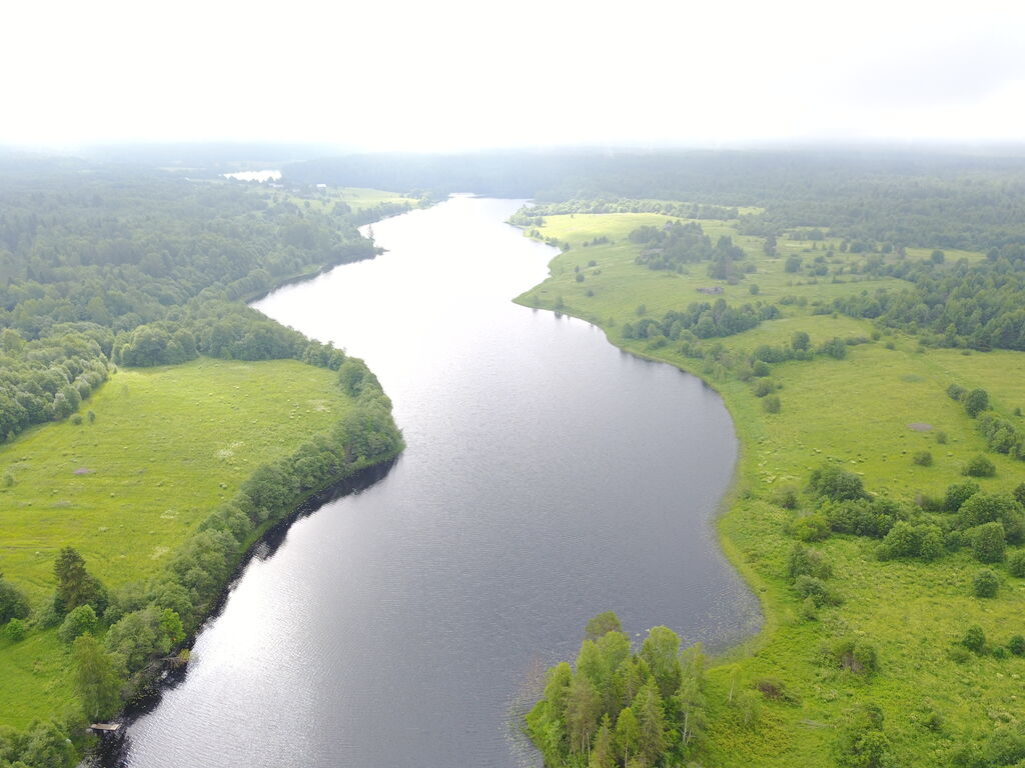
An example of a river-type lake, Dolgoe Lake (Vologda Region, Russia). Photo by Dmitriy A. Philippov (2018).

**Figure 5.**
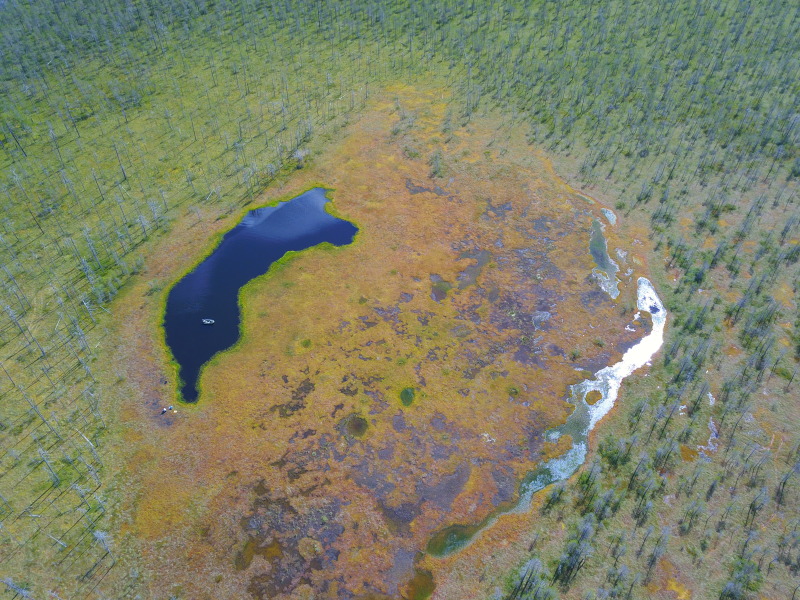
An example of a paludified lake, overgrown by floating mats, Ozeretskoe Lake (Vologda Region, Russia). Photo by Dmitriy A. Philippov (2020).

**Figure 6.**
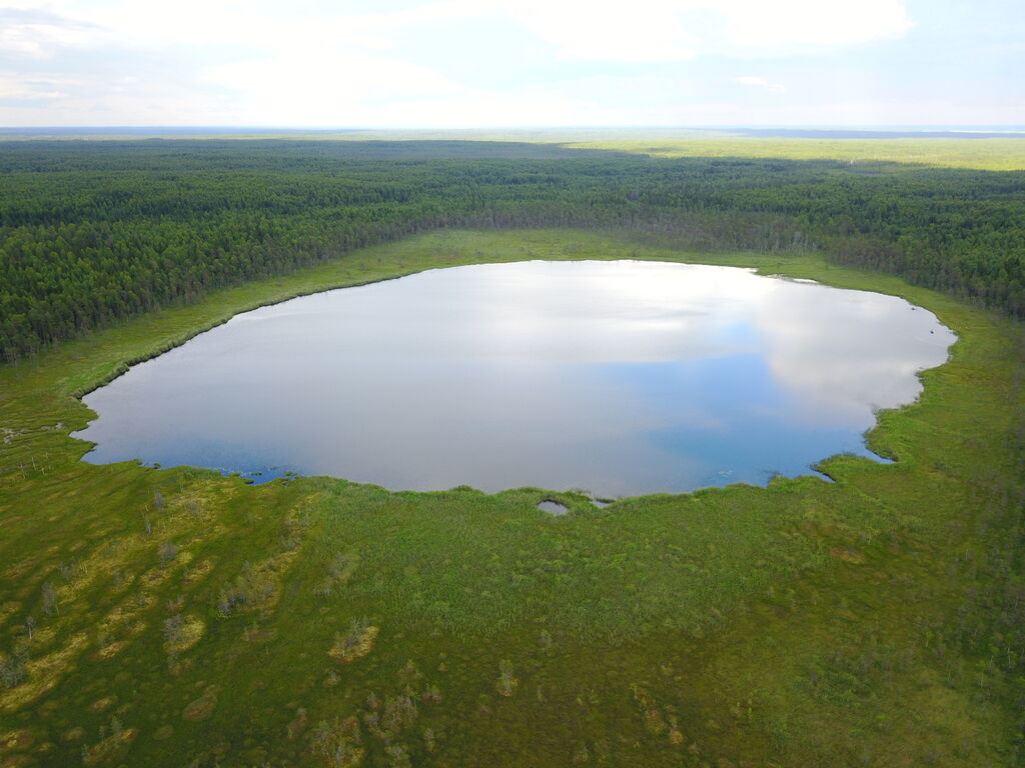
An intra-mire lake, Monozero Lake (Vologda Region, Russia). Photo by Dmitriy A. Philippov (2018).

**Figure 7.**
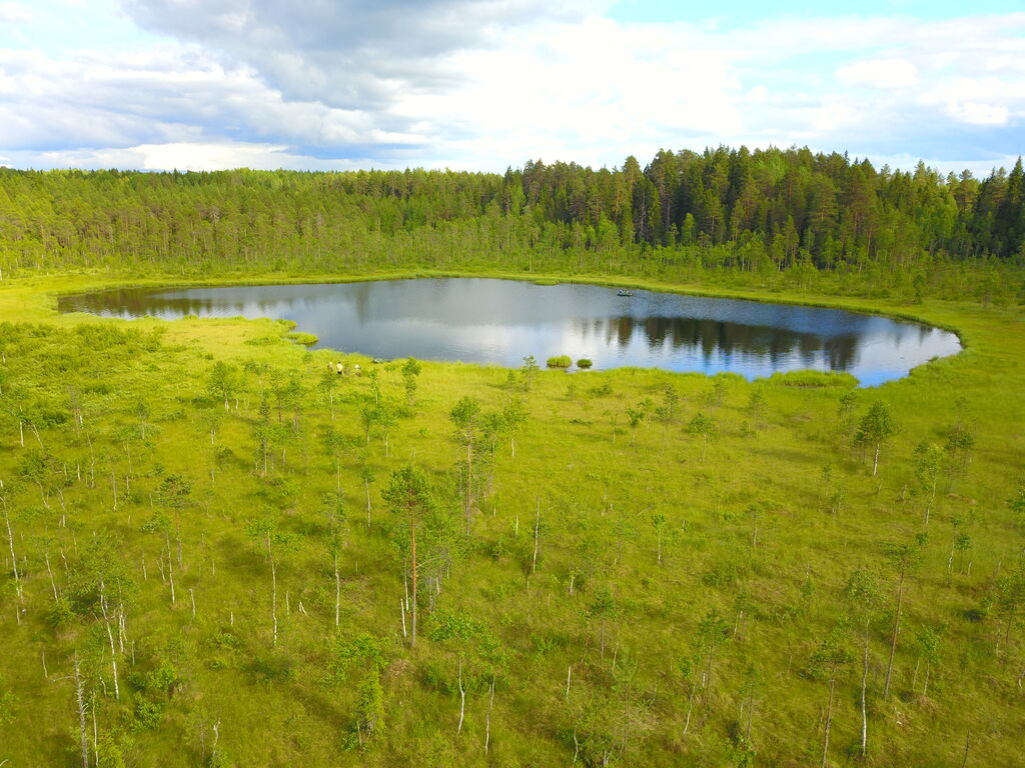
An intra-mire lake, Korovye Lake (Vologda Region, Russia). Photo by Dmitriy A. Philippov (2019).

**Figure 8.**
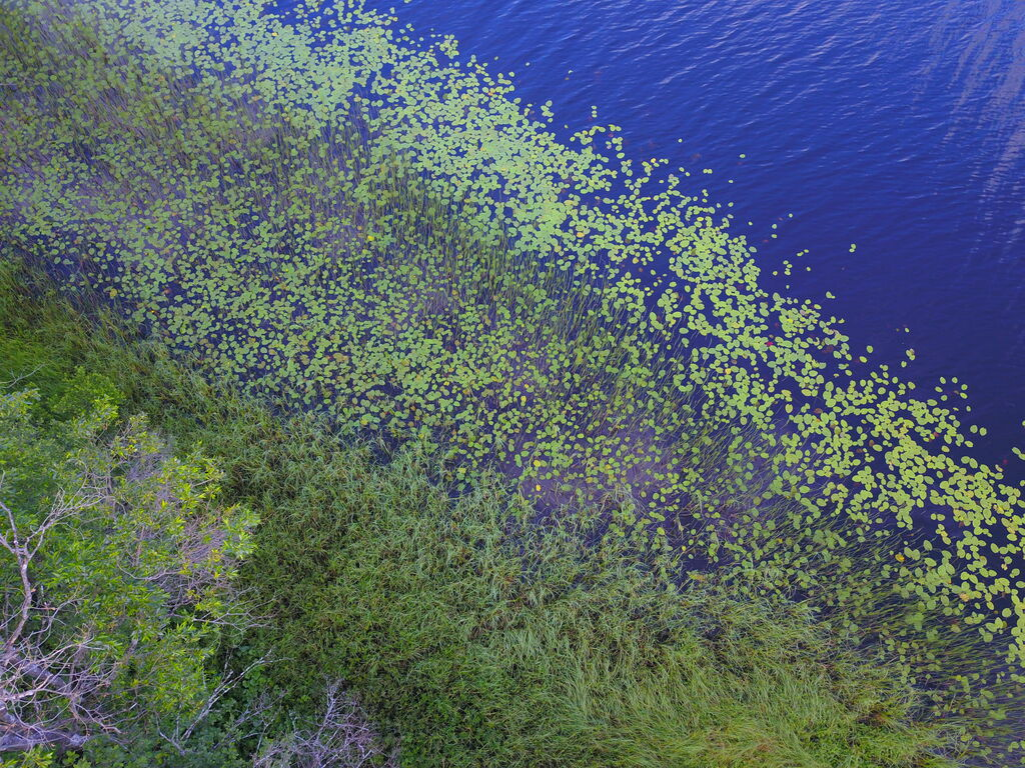
Belt-like overgrowth with *Menyanthestrifoliata*, *Carexrostrata*, *Equisetumfluviatile*, *Nupharlutea* in Svyatoe Lake (Vologda Region, Russia). Photo by Dmitriy A. Philippov (2019).

**Figure 9.**
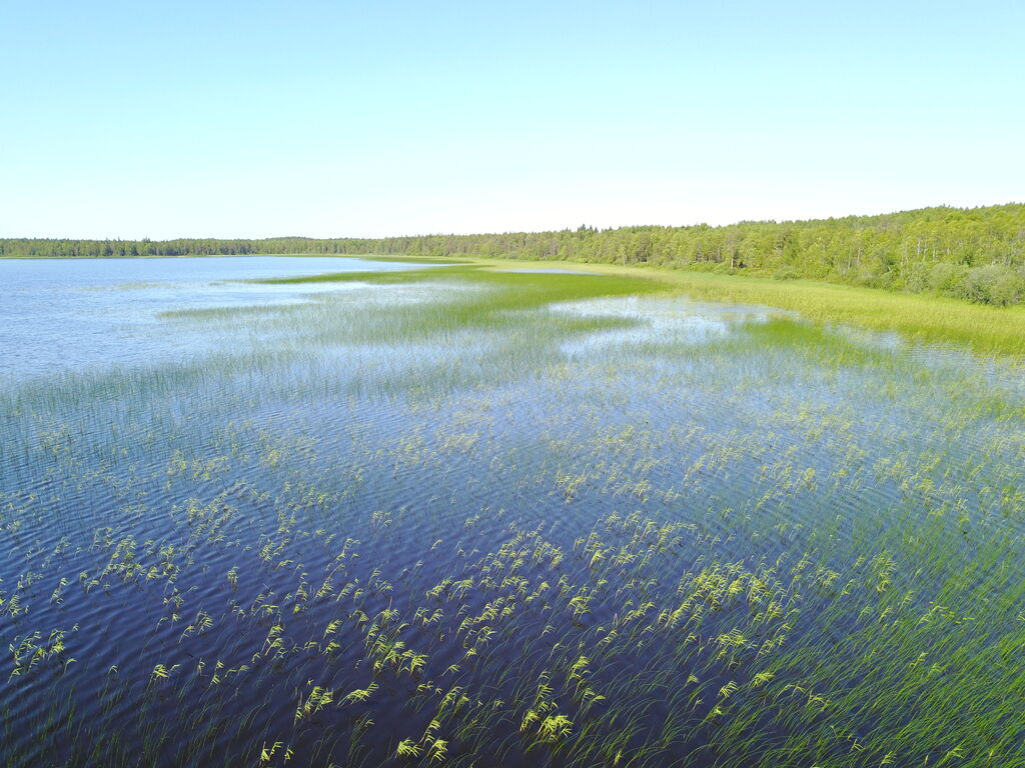
Helophyte communities (*Phragmitesaustralis* and *Schoenoplectuslacustris*) in Korgozero Lake (Vologda Region, Russia). Photo by Dmitriy A. Philippov (2018).

**Figure 10.**
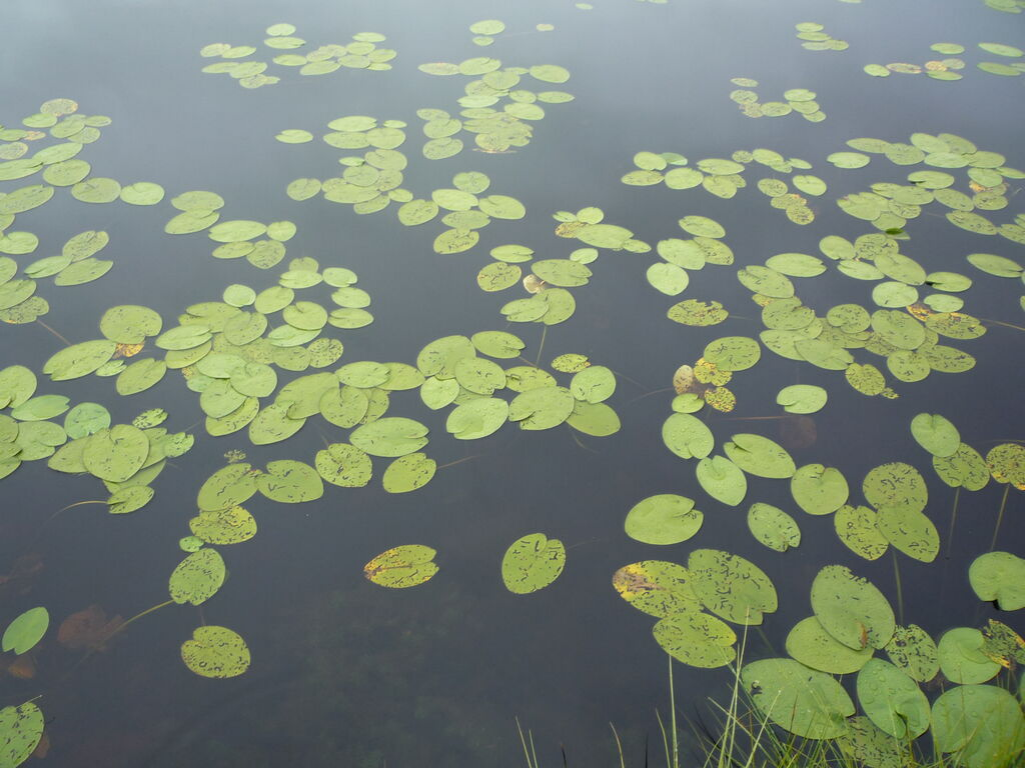
*Nupharlutea* communities in Chaykino Lake (Vologda Region, Russia). Photo by Dmitriy A. Philippov (2011).

**Figure 11.**
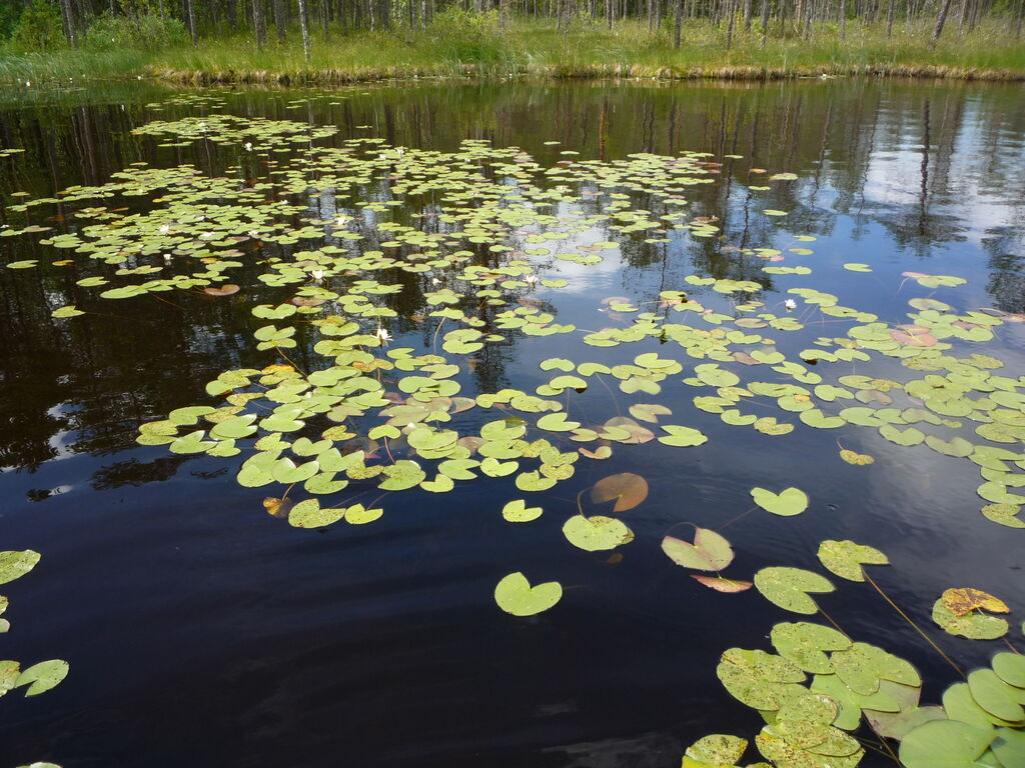
*Nymphaeacandida* communities in Borovskoe Lake (Vologda Region, Russia). Photo by Dmitriy A. Philippov (2019).

**Figure 12.**
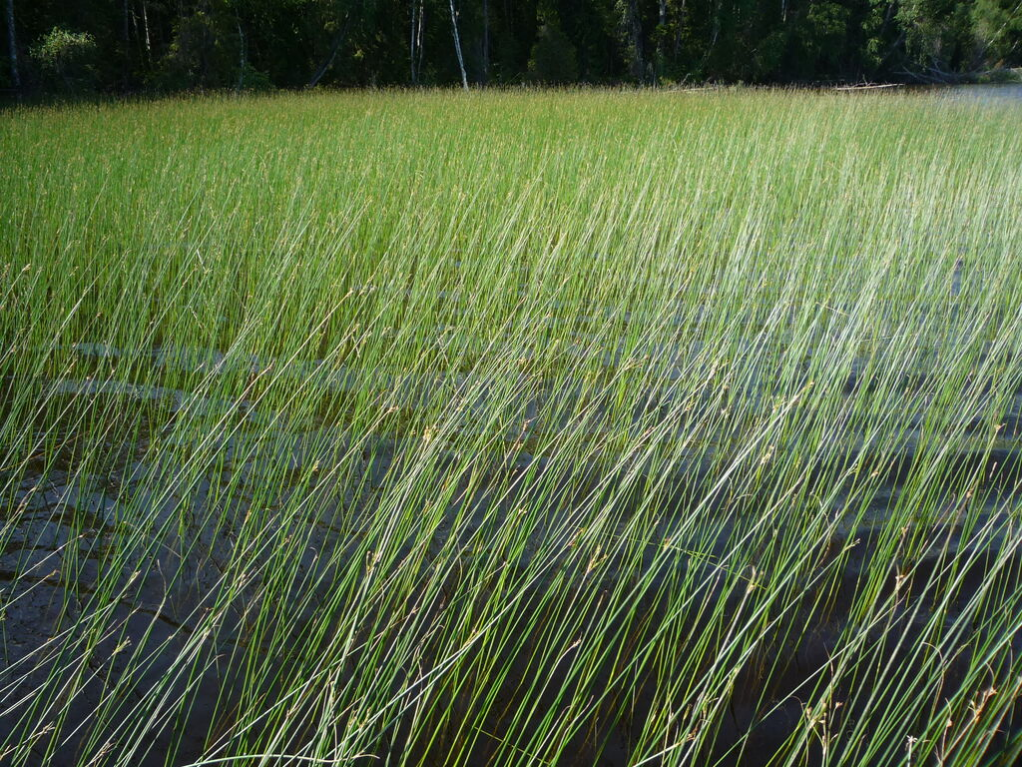
*Schoenoplectuslacustris* communities in Kovzhskoe Lake (Vologda Region, Russia). Photo by Dmitriy A. Philippov (2011).

**Figure 13.**
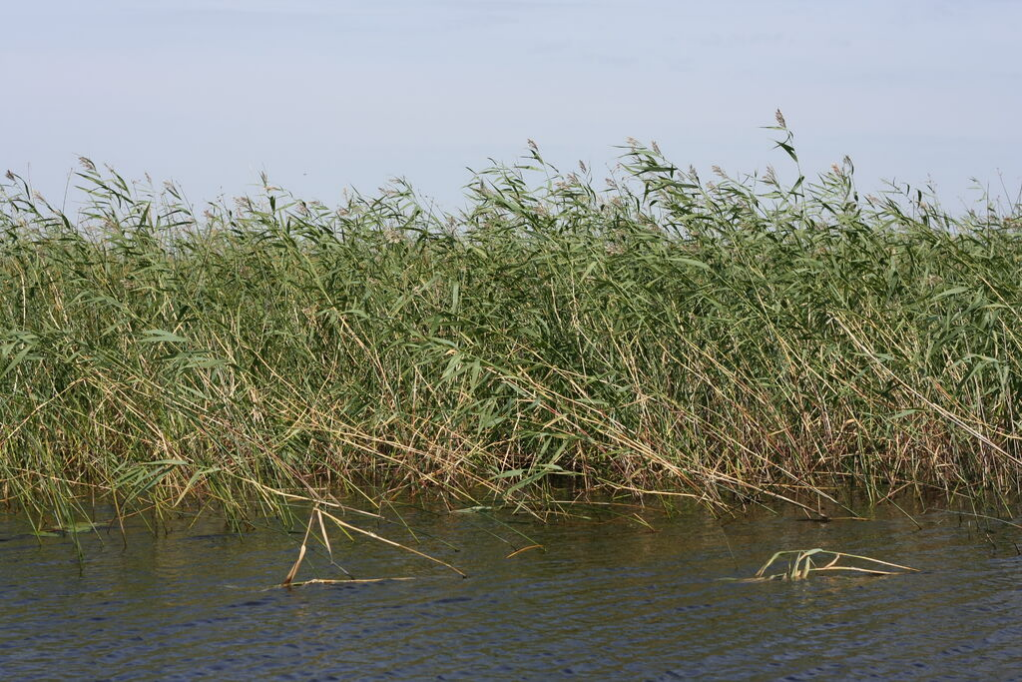
*Phragmitesaustralis* communities in Vozhe Lake (Vologda Region, Russia). Photo by Aleksandra S. Komarova (2018).

**Figure 14.**
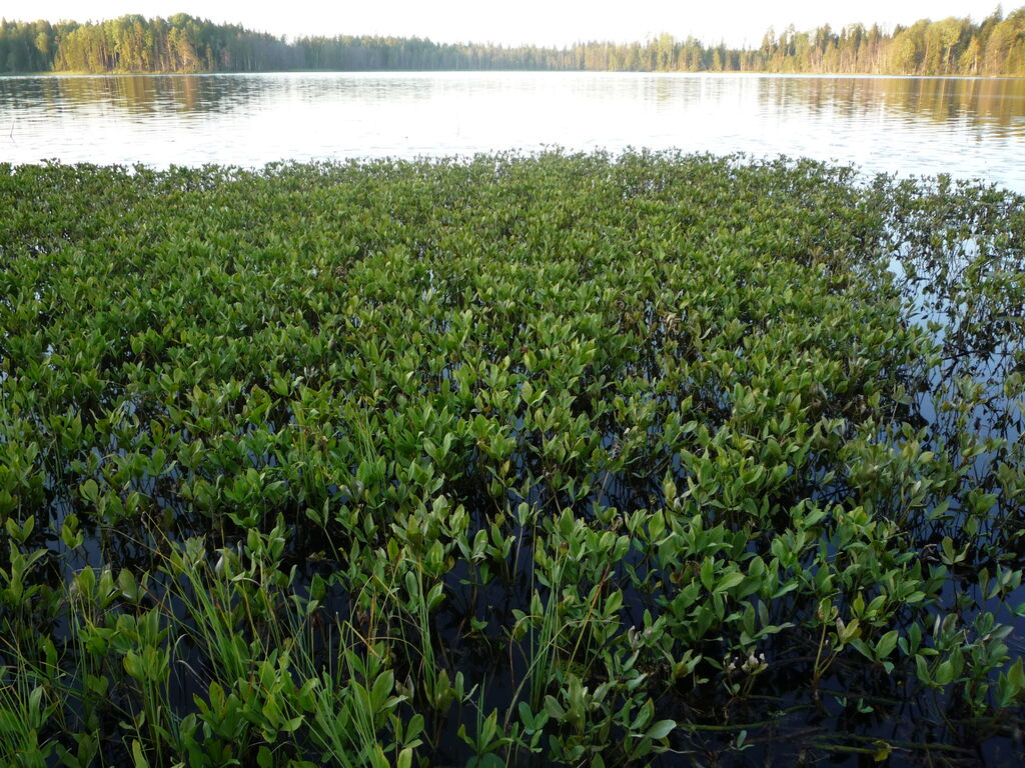
*Menyanthestrifoliata* communities in Laynozero Lake (Vologda Region, Russia). Photo by Dmitriy A. Philippov (2012).

**Table 1.** Aquatic organisms distribution (by number of occurrences) in different macrophyte communities in lakes of the Vologda Region.

Macrophyte communities	**Animalia**	**Bacteria**	**Chromista**	**Plantae**	**Protozoa**	**Total**
**Grand total**	**2205**	**137**	**666**	**288**	**27**	**3323**
**1. Aquatic plants**	**1009**	**81**	**432**	**193**	**22**	**1737**
**Macroalgae and aquatic mosses**	**395**					**395**
*Calliergonmegalophyllum*	19					19
*Charastrigosa*	7					7
*Fontinalisantipyretica*	243					243
*Scorpidiumscorpioides*	126					126
**Hydrophytes floating in the water**	**24**					**24**
*Stratiotesaloides*	24					24
**Submerged rooting hydrophytes**	**208**	**19**	**45**	**27**	**2**	**301**
*Elodeacanadensis*	45	4	13	4	1	67
*Potamogetongramineus*	69					69
*Potamogetonlucens*	38	15	30	20	1	104
*Potamogetonpectinatus*	9					9
*Potamogetonperfoliatus*	22					22
*Potamogetonpraelongus*	25		2	3		30
**Rooting hydrophytes with leaves floating on the water**	**357**	**44**	**311**	**118**	**9**	**839**
*Nupharlutea*	158	9	99	28	3	297
*Nupharlutea+Potamogetonnatans*	3	5	25	8	1	42
*Nymphaeacandida*	38	6	22	20		86
*Persicariaamphibia*	110	7	57	21	2	197
*Persicariaamphibia+Nupharlutea*		4	8	4	1	17
*Persicariaamphibia+Nymphaeacandida*	20		5	1		26
*Potamogetonnatans*	28	13	95	36	2	174
**Macroalgae and aquatic mosses and Submerged rooting hydrophytes**	**14**					**14**
*Elodeacanadensis+Fontinalisantipyretica*	14					14
**Hydrophytes floating in the water and Rooting hydrophytes with leaves floating on the water**		**1**	**21**	**4**	**3**	**29**
*Stratiotesaloides+Potamogetonnatans*		1	21	4	3	29
**Submerged rooting hydrophytes and Rooting hydrophytes with leaves floating on the water**	**11**	**17**	**55**	**44**	**8**	**135**
*Nupharlutea+Potamogetonlucens*	11	7	13	6	1	38
*Nupharlutea+Potamogetonperfoliatus*		10	42	38	7	97
**2. Semi-aquatic plants**	**625**	**30**	**98**	**44**	**5**	**802**
**Short-grass helophytes**	**102**					**102**
*Butomusumbellatus*	43					43
*Equisetumfluviatile*	9					9
*Sparganiumerectum*	50					50
**Tall-grass helophytes**	**281**	**26**	**74**	**36**	**4**	**421**
*Phragmitesaustralis*	157	24	54	32	2	269
*Phragmitesaustralis+Schoenoplectuslacustris*	9					9
*Schoenoplectuslacustris*	95	2	20	4	2	123
*Typhalatifolia*	20					20
**Hygrohelophytes**	**190**	**4**	**24**	**8**	**1**	**227**
*Carexrostrata*	50	4	24	8	1	87
*Carexrostrata+Menyanthestrifoliata*	36					36
*Eleocharispalustris*	78					78
*Menyanthestrifoliata*	26					26
**Short-grass helophytes and Hygrohelophytes**	**31**					**31**
*Equisetumfluviatile+Carexrostrata*	6					6
*Equisetumfluviatile+Menyanthestrifoliata*	25					25
**Tall-grass helophytes and Hygrohelophytes**	**21**					**21**
*Typhalatifolia+Carexrostrata+Menyanthestrifoliata*	21					21
**3. Coastal plants**	**351**					**351**
**Cryptogamic hygrophytes**	**351**					**351**
*Bryum* sp.	83					83
*Calliergon* sp.	80					80
*Calliergon* sp.+*Calliergonellacuspidata*	71					71
*Pseudobryumcinclidioides*	15					15
*Sphagnum* sp.	102					102
**4. Combinations**	**220**	**26**	**136**	**51**		**433**
**Aquatic plants and Semi-aquatic plants**	**155**	**23**	**128**	**41**		**347**
**Macroalgae and aquatic mosses and Tall-grass helophytes**	**19**					**19**
*Phragmitesaustralis+Charaaspera*	19					19
**Macroalgae and aquatic mosses and Hygrohelophytes**	**79**					**79**
*Carexrostrata+Fontinalisantipyretica*	49					49
*Carexrostrata+Menyanthestrifoliata+Fontinalisantipyretica*	30					30
**Rooting hydrophytes with leaves floating on the water and Short-grass helophytes**	**23**	**4**	**38**	**10**		**75**
*Equisetumfluviatile+Nupharlutea*		3	25	6		34
*Equisetumfluviatile+Nupharlutea+Nymphaeacandida+Potamogetonnatans*		1	13	4		18
*Equisetumfluviatile+Nymphaeacandida+Nupharlutea*	23					23
**Rooting hydrophytes with leaves floating on the water, Short-grass helophytes and Tall-grass helophytes**	**5**					**5**
*Phragmitesaustralis+Equisetumfluviatile+Nupharlutea*	5					5
**Rooting hydrophytes with leaves floating on the water, Short-grass helophytes and Hygrohelophytes**		**4**	**24**	**14**		**42**
*Equisetumfluviatile+Carexrostrata+Nupharlutea+Nymphaeacandida*		4	24	14		42
**Rooting hydrophytes with leaves floating on the water and Tall-grass helophytes**	**29**	**7**	**45**	**11**		**92**
*Phragmitesaustralis+Nupharlutea*	12	1	9	3		25
*Schoenoplectuslacustris+Nupharlutea*	4					4
*Schoenoplectuslacustris+Nymphaeacandida*	13	6	36	8		63
**Rooting hydrophytes with leaves floating on the water and Hygrohelophytes**		**8**	**21**	**6**		**35**
*Carexrostrata+Nymphaeacandida*		3	7	4		14
*Menyanthestrifoliata+Nymphaeacandida*		5	14	2		21
**Aquatic plants, Semi-aquatic plants and Coastal plants**	**25**	**3**	**8**	**10**		**46**
**Submerged rooting hydrophytes, Hygrohelophytes and Cryptogamic hygrophytes**	**15**					**15**
*Carexrostrata+Myriophyllumverticillatum+Calliergon* sp.	15					15
**Rooting hydrophytes with leaves floating on the water, Hygrohelophytes and Herbaceous hygrophytes**	**10**	**3**	**8**	**10**		**31**
*Carexlasiocarpa+Menyanthestrifoliata+Nupharlutea*		3	8	10		21
*Carexlasiocarpa+Menyanthestrifoliata+Nymphaeacandida*	10					10
**Aquatic plants and Coastal plants**	**5**					**5**
**Submerged rooting hydrophytes and Cryptogamic hygrophytes**	**5**					**5**
*Sphagnum* sp.+*Utriculariaintermedia*	5					5
**Semi-aquatic plants and Coastal plants**	**35**					**35**
**Hygrohelophytes and Cryptogamic hygrophytes**	**21**					**21**
*Carexrostrata+Calliergon* sp.	21					21
**Hygrohelophytes and Herbaceous hygrophytes**	**14**					**14**
*Carexlasiocarpa+Menyanthestrifoliata*	14					14
